# Intramolecular hydroamination of alkynic sulfonamides catalyzed by a gold–triethynylphosphine complex: Construction of azepine frameworks by 7-*exo*-*dig* cyclization

**DOI:** 10.3762/bjoc.7.106

**Published:** 2011-07-08

**Authors:** Hideto Ito, Tomoya Harada, Hirohisa Ohmiya, Masaya Sawamura

**Affiliations:** 1 Department of Chemistry, Faculty of Science, Hokkaido University, Sapporo 060-0810, Japan

**Keywords:** azepine, cyclization, gold catalyst, hydroamination, triethynylphosphine

## Abstract

The gold-catalyzed, seven-membered ring forming, intramolecular hydroamination of alkynic sulfonamides has been investigated. The protocol, with a semihollow-shaped triethynylphosphine as a ligand for gold, allowed the synthesis of a variety of azepine derivatives, which are difficult to access by other methods. Both alkynic sulfoamides with a flexible linear chain and the benzene-fused substrates underwent 7-*exo*-*dig* cyclization to afford the nitrogen-containing heterocyclic seven-membered rings, such as tetrahydroazepine and dihydrobenzazepine, in good yields.

## Introduction

**Figure 1:**
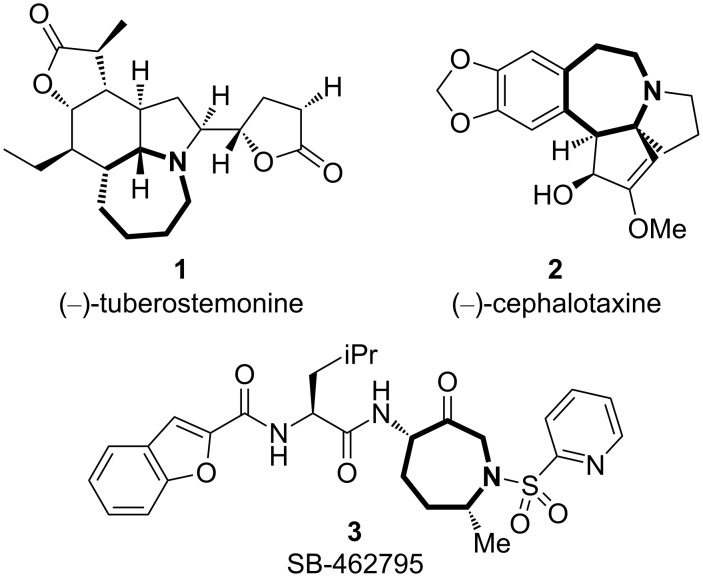
Azepine frameworks found in natural products and pharmaceuticals.

Nitrogen-containing heterocyclic seven-membered rings are found in many biologically active natural products and pharmaceuticals, such as (−)-tuberosutemonin (**1**) [1–6], related *Stemona* alkaloids [7], *Cephalotaxus* alkaloid (−)-cephalotaxine (**2**) [8–12], and SB-462795 (**3**) (Figure 1) [13–16]. Among a number of different approaches for the construction of N-heterocyclic compounds, metal-catalyzed intramolecular hydroamination of unactivated C–C multiple bonds is particularly straightforward and efficient [17–18]. Specifically, gold-catalyzed intramolecular hydroaminations of alkynes, alkenes and allenes show remarkable efficiency [19–28]. Unfortunately, however, the application of these methodologies to the synthesis of the N-heterocyclic seven-membered ring compounds is hampered by the low efficiency of seven-membered ring formations. Despite extensive studies on the gold-catalyzed intramolecular hydroamination of alkynes [19–51], seven-membered ring formation is rare and is limited to the cases where the substrate is preorganized for cyclization: The substrates must have geminal disubstitution or ring fusion within a linker chain connecting the attacking nitrogen atom and the alkyne moiety. It should be noted, however, that 7-“*endo”*-*dig* cyclizations of (*o*-alkynyl)phenylacetamides and a diynamide were achieved with gold and palladium complexes [39,46,52–53], and the zinc-catalyzed 7-*exo*-*dig* cyclization was reported specifically for a propargyl ether substrate [54].

**Figure 2:**
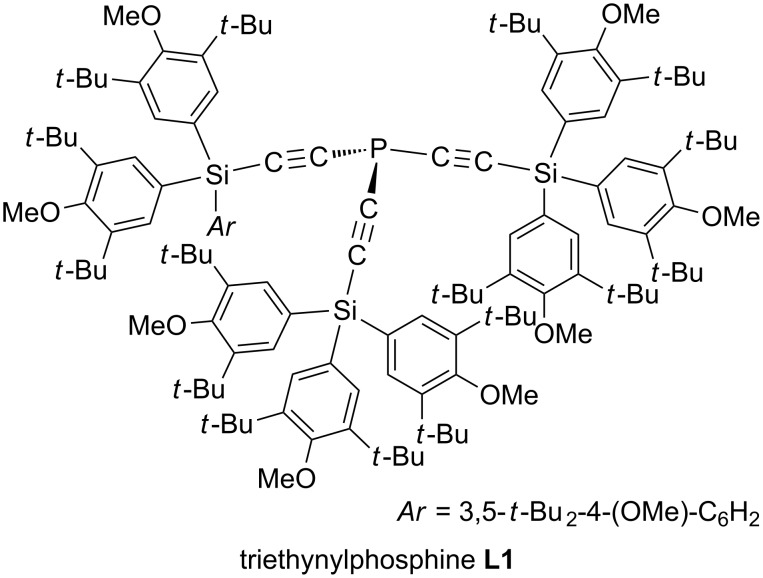
Semihollow-shaped triethynylphosphine **L1**.

Previously, we reported that semihollow-shaped triethylnylphosphine **L1** (Figure 2) exerted marked acceleration effects in the gold(I)-catalyzed Conia-ene reactions of acetylenic keto esters and enyne cycloisomerizations. The new catalytic system has expanded the scope of the reactions to six- and seven-membered ring formations, which had been difficult with the conventional catalytic systems [55]. Furthermore, we found that **L1**–gold(I) complex efficiently catalyzed the cyclization of internal alkyne substrates, which had also been difficult due to the steric repulsion between a nucleophilic center and a terminal substituent on the alkyne moiety [56]. We proposed that the cavity in the ligand forces the nucleophilic center closer to the gold-bound alkyne, resulting in the entropy-based rate enhancement. Recently, we further developed the gold(I)-catalyzed 7-*exo*-*dig* cyclization of acetylenic silyl enol ethers with **L1** [57].

In this context, we expected that the use of **L1** as a ligand in the gold-catalyzed intramolecular hydroamination of alkynes would enable the construction of nitrogen-containing heterocyclic seven-membered rings, and we applied the triethynylphosphine–gold(I) catalytic systems to the synthesis of azepine derivatives through intramolecular hydroamination of alkynic sulfonamides. This article describes the results of the optimization of reaction conditions, exploration of substrate scope, and some mechanistic experiments.

## Results and Discussion

### Reaction conditions

The reaction conditions were optimized for the cyclization of *N*-(6-heptyn-1-yl)-*p*-toluenesulfonamide (**4a**) (Table 1). The triethynylphosphine–gold complex [Au(NTf_2_)(**L1**)] (0.5 mol %) catalyzed the cyclization of **4a** (0.2 mmol) efficiently in CH_2_Cl_2_ (1.0 mL) at 25 °C (100% convn. of **4a**) to afford 4,5,6,7-tetrahydroazepine derivative **5a** with 18 h reaction time in 82% isolated yield (Table 1, entry 1). This reaction seemed to proceed through 7-*exo*-*dig* cyclization, but an exomethylene-type cyclic product **6a**, which is a possible product of the 7-*exo*-*dig* cyclization [57], was not observed. The reaction under four-times-diluted conditions did not proceed to full conversion in the same reaction time (Table 1, entry 2). The reaction time was shortened to 9 h by heating at 80 °C, but this caused a slight decrease in the isolated yield of **5a** (79%) (Table 1, entry 3).

**Table 1:** Optimization of reaction conditions for the gold-catalyzed intramolecular hydroamination of **4a**.

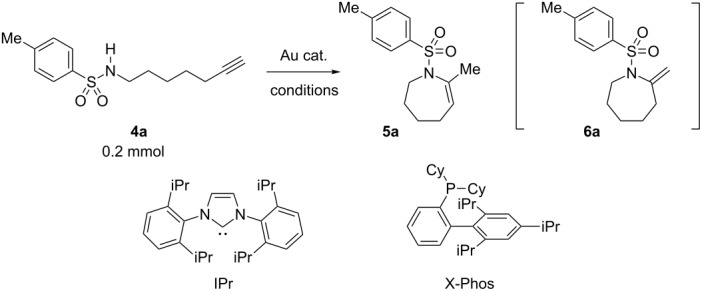						

entry	Au cat. (mol %)	solvent (mL)	temp. (°C)	time (h)	convn. (%)^a^	yield orf **5** (%)^a,b^

1	[Au(NTf_2_)(**L1**)] (0.5)	CH_2_Cl_2_ (1.0)	25	18	100	90 (82)
2	[Au(NTf_2_)(**L1**)] (0.5)	CH_2_Cl_2_ (4.0)	25	18	87	85
3	[Au(NTf_2_)(**L1**)] (0.5)	DCE (1.0)	80	9	100	83 (79)
4	[Au(NTf_2_)(**L1**)] (0.5)	toluene (1.0)	25	18	98	97
5	[Au(NTf_2_)(**L1**)] (0.5)	THF (1.0)	25	18	29	24
6	[Au(NTf_2_)(**L1**)] (0.5)	MeCN (1.0)	25	18	0	n. d.
7	[Au(OTf)(**L1**)] (0.5)	CH_2_Cl_2_ (1.0)	25	18	100	90 (80)
8	[Au(SbF_6_)(**L1**)] (0.5)	CH_2_Cl_2_ (1.0)	25	18	14	n. d.
9	[Au(BF_4_)(**L1**)] (0.5)	CH_2_Cl_2_ (1.0)	25	18	7	n. d.
10	[Au(NTf_2_)(PPh_3_)] (0.5)	CH_2_Cl_2_ (1.0)	25	24	29	22 (26)
11	[Au(NTf_2_)(PPh_3_)] (5.0)	CH_2_Cl_2_ (1.0)	25	18	100	66 (64)
12	[Au(NTf_2_){P(OPh)_3_}] (0.5)	CH_2_Cl_2_ (1.0)	25	18	54	39
13	[Au(NTf_2_)(X-Phos)] (0.5)	CH_2_Cl_2_ (1.0)	25	18	41	41
14	[Au(NTf_2_)(IPr)] (0.5)	CH_2_Cl_2_ (1.0)	25	18	53	41

^a^Determined by ^1^H NMR. ^b^Isolated yield in parentheses.

Among other solvents examined, toluene gave a result comparable with CH_2_Cl_2_ (Table 1, entries 1 and 4). On the other hand, polar and potentially coordinating solvents such as THF and MeCN were not effective in this reaction (Table 1, entries 5 and 6). The effect of the counter anion of the cationic gold complex is shown in Table 1, entries 1 and 7–9. While OTf^−^ was as effective as NTf_2_^−^ (Table 1, entry 7), SbF_6_^−^ and BF_4_^−^ inhibited the reaction completely (Table 1, entries 8 and 9).

The ligand effect is evaluated in Table 1, entries 1 and 10–14. The reaction proceeded slowly, even with a conventional phosphine ligand PPh_3_, such that the starting material was not fully consumed even after 24 h and the yield was as low as 26% (Table 1, entry 10). Increasing the catalyst loading of [Au(NTf_2_)(PPh_3_)] to 5.0 mol % caused full consumption of **4a**, but the cyclization product was obtained only in 64% yield (Table 1, entry 11). The low yield relative to the conversion value is probably due to oligomerization and/or product decomposition as suggested by TLC and ^1^H NMR analysis of the crude mixture. A phosphite ligand P(OPh)_3_, which is comparable to triethynylphosphine **L1** in electron-donor ability [58], was slightly more effective than PPh_3_, but was far less effective than **L1** (Table 1, entry 12). Bulky and strongly electron-donating ligands such as X-Phos and IPr were as effective as the electron-deficient ligand P(OPh)_3_ (Table 1, entries 13 and 14). Accordingly, it is concluded that the acceleration effect by **L1** is not due to an electronic effect rather a steric effect.

**Figure 3:**
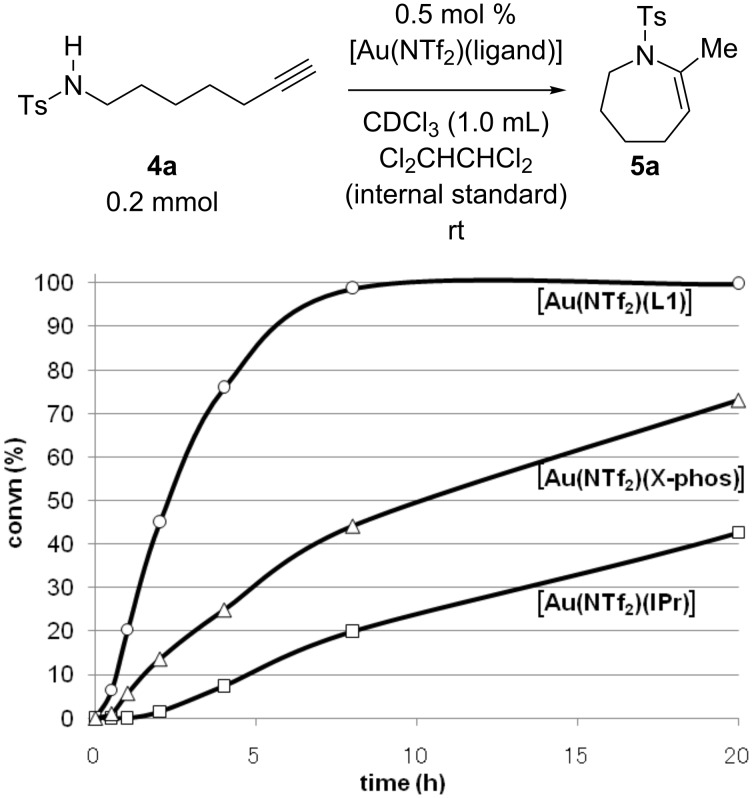
Time–conversion profiles for the gold-catalyzed cyclization of **4a** with **L1**, X-Phos and IPr ligands.

The time–conversion profiles shown in Figure 3 clearly indicate that the high catalytic efficiency with **L1** is due to the improvement of the reaction kinetics and not the thermal stability of the catalyst. Although it was reported that Au(NTf_2_)(IPr) was somewhat unstable in the gold-catalyzed intermolecular hydroamination of alkyne under heating conditions [59], the deactivation of the gold catalyst with IPr and X-Phos was not significant under the present reaction conditions: The reactions with X-Phos and IPr ligands reached 100% and 84% conversions after 58 h, respectively (see Supporting Information File 1 for reaction profiles with longer reaction times).

### Effect of N-substituents

While alkynic *o*-nitrotoluenesulfonamide **4b** did not react at all with 0.5 mol % of [Au(NTf_2_)(**L1**)] at room temperature (Table 2, entry 1), this substrate underwent 7-*exo*-*dig* cyclization upon increasing catalyst loading to 2.5 mol % and heating at 80 °C, giving *N*-nosylazepine derivative **5b** in 76% isolated yield (Table 2, entry 2). *N*-Benzyloxycarbonyl (Cbz) and *N*-acetylazepine derivatives **5c** and **5d** were obtained in low yields through the cyclization of substrates **4c** and **4d** (Table 2, entries 3 and 4). On the other hand, the reactions of the substrates bearing *N*-*tert*-butoxycarbonyl (Boc) or *N*-*p*-methoxybenzyl (PMB) groups (**4e**,**f**) did not give the desired products at all (Table 2, entries 5 and 6). It seems that the reactivity of the substrates is affected by the balance between nucleophilicity of the nitrogen atom and acidity of the N–H bond as well as a steric factor.

**Table 2:** Effect of N-substitutents.

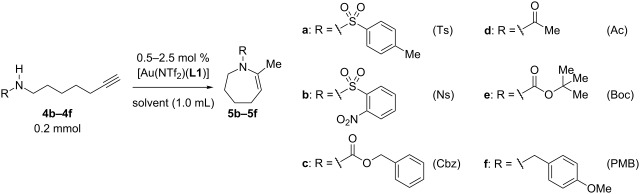							

entry	R	Au cat. (mol %)	solvent	temp. (°C)	time (h)	convn. (%)^a^	yield of **5** (%)^a^

1	Ns (**4b**)	0.5	CH_2_Cl_2_	25	18	0	n. d.
2	Ns (**4b**)	2.5	DCE	80	18	100	76^b^
3	Cbz (**4c**)	2.5	DCE	80	24	97	33
4	Ac (**4d**)	2.5	DCE	80	24	63	18
5	Boc (**4e**)	2.5	DCE	80	24	17	n. d.
6	PMB (**4f**)	2.5	DCE	80	24	0	n. d.

^a^Determined by ^1^H NMR. ^b^Isolated yield.

### Effect of substituents in acyclic linkers

Next, we explored the substrate scope by introducing one or two substituents in the acyclic linker chain of the alkynic *N*-tosylsulfonamide **4a** (Table 3). The introduction of the substituents at the α or β positions relative to the alkyne moiety caused a significant decrease in the reactivity, but the cyclization of the substituted alkynic sulfonamide **4g**–**l** proceeded smoothly, with 2.5–5.0 mol % catalyst loading at 80 °C, into full substrate conversion. Specifically, the substrate bearing an α-Me group (**4g**) derived from L-alanine was quantitatively converted into 2,7-dimethyltetrahydroazepine **5g** with 2.5 mol % of [Au(NTf_2_)(**L1**)] (99% isolated yield, Table 3, entry 1). Although the substitution with bulkier iPr or Bn groups at the α-position in **4h** and **4i** resulted in even lower reactivities, the corresponding cyclization products **5h** and **5i** were obtained in high or good yields (Table 3, entries 2 and 3). The substrates (**4j**–**l**) with geminal disubstitution at the β-carbon also participated in the 7-*exo*-*dig* cyclization in good yields (Table 3, entries 4–6). Among the cyclization products (**5a**–**l**) described above, only the β,β-diphenyl-substituted sulfonamide **5k** was contaminated with a small amount of exomethylene product **6k** (Table 3, entry 5).

It should be noted that the geminal disubstitution in **4j**–**l** caused a drastic decrease in the ease of the cyclization, which necessitated much more harsh reaction conditions (5 mol % Au, 80 °C, 4–12 h, Table 3, entries 4–6) than those for the reaction of the parent substrate **4a** (0.5 mol % Au, 25 °C, 18 h, Table 1, entry 1). This means that the Thorpe–Ingold effect did not operate in the present case and that the substituents caused steric repulsion hindering the cyclization.

**Table 3:** Cyclization of alkynic sulfonamides with an acyclic linker.^a^

entry	substrate	Au cat. (mol %)	time (h)	convn. (%)^b^	product	yield (%)^c^

	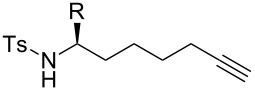				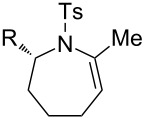	
1	R = Me (**4g**)	2.5	8	100	R = Me (**5g**)	99
2	R = iPr (**4h**)	5.0	12	100	R = iPr (**5h**)	88
3	R = Bn (**4i**)	5.0	12	100	R = Bn (**5i**)	71
	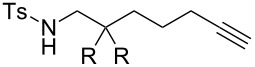				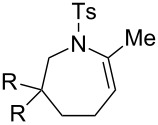	
4	R = Me (**4j**)	5.0	4	100	R = Me (**5j**)	77
5	R = Ph (**4k**)	5.0	4	100	R = Ph (**5k**)	69^d^
6	R = –(CH_2_)_5_– (**4l**)	5.0	12	100	R = –(CH_2_)_5_– (**5l**)	66

^a^Reaction conditions: **4**, 0.1 mmol; [Au(NTf_2_)(**L1**)], 2.5 or 5 mol %; DCE, 1.0 mL; 80 °C. ^b^Determined by ^1^H NMR. ^c^Isolated yield. ^d^Mixture of **5k** and **6k** in 92:8 ratio. 
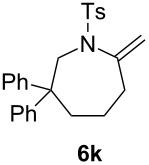

### Construction of bicyclic frameworks

Next, we applied the gold(I)–triethynylphosphine **L1** complex to the construction of bicyclic frameworks such as benzazepine (Table 4). The cyclization of *o*-alkynyl benzylsulfonamide **4m** proceeded with both [Au(NTf_2_)(**L1**)] and [Au(NTf_2_)(IPr)] to give a benzazepine derivative **5m** (Table 4, entries 1 and 2). Although the starting material was fully consumed after 3 h or 6 h, using the respective catalysts, **L1** was superior to IPr with respect to both reaction time and product yield. The reaction of *N*-tosylbenzamide **4n** with **L1** afforded the benzene-fused ε-caprolactam **6n** within an exomethylene structure in 97% yield in an isomerically pure form (vide infra for discussion) (Table 4, entry 3). Sulfonamide **4o**, with a cyclohexane-fused linker, also participated in the cyclization to form azabicylo[5.4.0]decene **5o** in 76% yield along with a small amount of exomethylene isomer **6o** (**5o**/**6o** 98:2, Table 4, entry 4).

**Table 4:** Cyclization of alkynic sulfonamide with a ring-fused linker.^a^

entry	substrate	Au cat. (mol %)	time (h)	convn. (%)^b^	product	yield (%)^c^

1	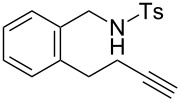 **4m**	[Au(NTf_2_)(**L1**)] (2.5)	3	100	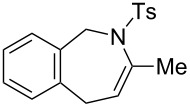 **5m**	86
2	**4m**	[Au(NTf_2_)(IPr)] (2.5)	6	100	**5m**	58
3	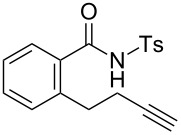 **4n**	[Au(NTf_2_)(**L1**)] (5.0)	3	100	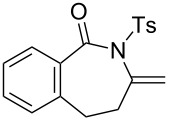 **6n**	97
4	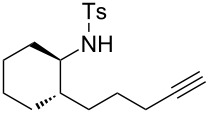 **4o**	[Au(NTf_2_)(**L1**)] (2.5)	17	100	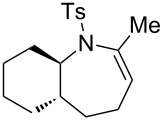 **5o**	76^d^

^a^Reaction conditions: **4**, 0.1 mmol; DCE, 1.0 mL; 80 °C. ^b^Determined by ^1^H NMR. ^c^Isolated yield. ^d^Mixture of **5o** and **6o** in 98:2 ratio. 
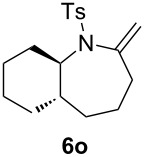

### Effect of ring sizes

We also evaluated the triethynylphosphine **L1**, X-Phos, and IPr for an acceleration effect in the six-membered, ring forming, gold-catalyzed hydroamination of *N*-(5-hexyn-1-yl)-*p*-toluenesulfonamide **7**. As expected from entropy considerations, the six-membered ring formations of **7** with these ligands were generally much faster than the seven-membered ring formations of **4**: The reaction with 0.5 mol % catalyst loading at room temperature completed within 1 h irrespective of the ligand used. When the catalyst loading was reduced to 0.1 mol %, however, the superiority of **L1** to X-Phos and IPr became significant, as shown in Table 5. The reaction with **L1** at room temperature afforded the six-membered ring product **8** in 91% isolated yield after 2 h (Table 1, entry 1). On the other hand, the reaction with X-Phos did not reach full conversion (76% convn.) even after 12 h and gave **8** in only 70% yield (Table 5, entry 2). The use of IPr ligand resulted in even lower conversion (68%) and isolated yield (58%) (Table 5, entry 3).

**Table 5:** 6-*exo-dig* cyclization of sulfonamide **7**.

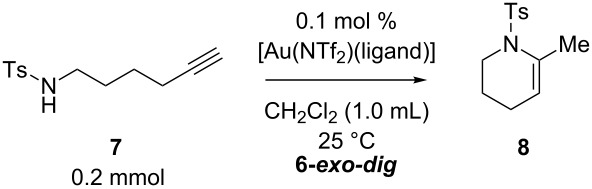				

entry	Ligand	time (h)	convn. (%)^a^	yield of **8** (%)^a,b^

1	**L1**	2	100	100 (91)
2	X-Phos	12	76	76 (70)
3	IPr	12	68	67 (58)

^a^Determined by ^1^H NMR. ^b^Isolated yield in the parentheses.

**Scheme 1:**
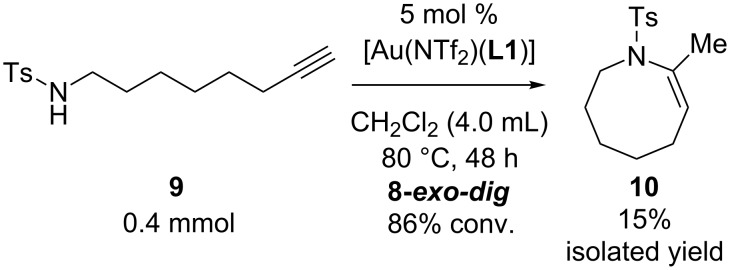
8-*exo-dig* cyclization of sulfonamide **9**.

The triethynylphosphine ligand **L1** was also evaluated for the eight-membered ring formation of sulfonamide **9**, which is much more challenging than the seven-membered ring formation of **4**. The reaction required 5 mol % catalyst loading under heating conditions (80 °C) in 1,2-dichloroethane for a reasonable conversion rate to afforded an eight-membered ring azocine derivative **10** in 15% isolated yield (Scheme 1). It should be noted that the reaction produced significant amounts of unidentified oligomeric side products.

### Alkene isomerization

**Scheme 2:**
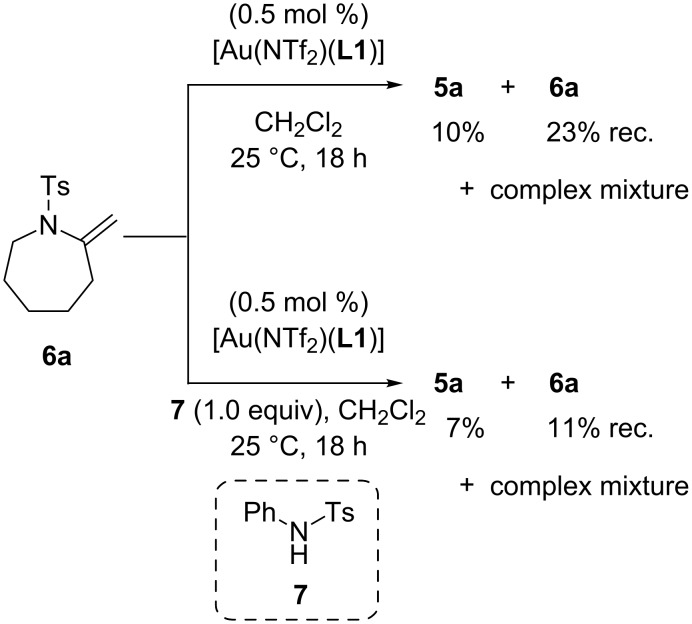
Isomerization experiments of **6a**.

We carried out alkene isomerization experiments to clarify how tetrahydroazepines **5** formed via the 7-*exo*-*dig* cyclization of **4**. One possible reaction pathway is the alkene isomerization of an exomethylene product **6**. To test this possibility, we synthesized **6a** through another route (see Supporting Information File 1) and subjected it to the standard reaction conditions of the gold–triethynylphosphine-catalyzed cyclization of alkynic sulfonamide with or without *N*-tosyl aniline **7** as an external proton source (Scheme 2). Although **6a** was indeed isomerized into **5a** to some extent in both cases, the main reaction was decomposition to give complex mixtures. The exomethylene substrate **6a** appeared to be unstable at room temperature even in the absence of the gold-catalyst.

**Figure 4:**
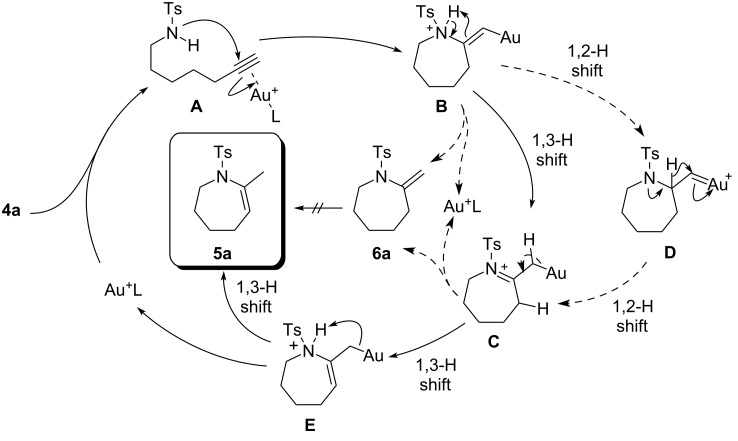
Possible pathway for the gold-catalyzed conversion of **4a** into **5a**.

According to these results, the formation of exomethylene compound **6** and subsequent alkene isomerisation should not be a major pathway to **5**. Instead, a possible reaction pathway from **4a** to **5a** is illustrated in Figure 4. First, the cationic gold center coordinates with **4a** to form the gold–alkyne complex **A**. Intramolecular nucleophilic attack of the nitrogen atom affords the 7-*exo*-*dig* cyclization product **B** with an exocyclic C–C double bond. The protonated *N*-sulfonylenamide **B** tautomerizes to iminium ion **C** through 1,3-proton shift, or through an alternative pathway via a gold(I)–carben intermediate (**D**). Then, re-tautomerization affords the protonated *N*-sulfonylenamide **E** with an endocyclic C–C double bond. Finally, protodemetalation of **E** give the *N*-sulfonylenamide **5a**, which is thermodynamically more stable than **6a**.

It should be noted that the reaction of the *N*-tosylbenzamide **4n** afforded exceptionally the exomethylene isomer **6n**. One conceivable reason is that the alkene isomerisation was prevented due to a ring strain in the seven-membered ring of **5n**, of which six out of seven atoms are sp^2^-hybridized.

## Conclusion

We demonstrated that the 7-*exo*-*dig* intramolecular hydroamination of ω-alkynic *N*-alkyl-*N*-sulfonamides is efficiently catalyzed by a gold(I) complex coordinated with the semihollow-shaped triethynylphopshine ligand **L1**, and that the cyclization protocol provides a new efficient route to N-containing seven-membered ring compounds. The protocol is applicable to the reaction of alkynic sulfonamides with an acyclic or ring-fused linker chain with various substitution patterns. Evaluation of the ligand effect in the gold catalysis with different ligands and substrates strongly suggested that the rate enhancement by the triethynylphosphine would be due to a steric factor which enforces a nucleophilic center close to a gold-activated alkyne moiety.

## Experimental

### Preparation of [Au(NTf_2_)(L1)]

[AuCl(**L1**)] (1 equiv) was placed in an open vial and was dissolved in CH_2_Cl_2_ (ca. 0.1 M). AgNTf_2_ (>1.5 equiv) was added, and the mixture was stirred at 25 °C for 10 min. The resulting white suspension was filtered through celite into a screw vial. The resulting colorless solution was first concentrated with a stream of Ar gas and then dried in vacuo to give [Au(NTf_2_)(**L1**)] as a white solid. (See also [55].)

### General procedure for gold-catalyzed intramolecular hydroamination of alkynic sulfonamide **4a**

[Au(NTf_2_)(**L1**)] (2.6 mg, 1.0 μmol, 0.5 mol %) and a magnetic stirring bar were placed in an open vial. Separately, the alkynyl sulfonamide **4a** (55 mg, 0.20 mmol) was weighted into a micro tube. The tubes were placed in a glove box. The gold complex and **4a** were dissolved in degassed dry CH_2_Cl_2_ (0.25 mL), in their respective tubes. The solution of **4a** was transferred to the solution of the catalyst with a syringe. The remaining solutions in the micro tube and the syringe were washed with CH_2_Cl_2_ (2 × 0.25 mL) and, the washings were added to the reaction mixture. The tube was sealed with a cap equipped with a Teflon-coated silicon rubber septum. The tube was taken from the glove box, and was placed in a water bath (25 °C). After the reaction was complete (as monitored by TLC), the reaction mixture was passed through a pad of silica gel and was concentrated to dryness. Purification by flash chromatography on silica gel gave the cyclization product **5a** (45 mg, 82%) as a white solid; mp 65.8–66.1 °C; ^1^H NMR (300 MHz, CDCl_3_) δ 1.32–1.53 (m, 6H), 1.94 (t, *J* = 2.7 Hz, 1H), 2.14 (td, *J* = 6.9, 2.7 Hz, 2H), 2.44 (s, 3H), 2.95 (q, *J* = 6.9 Hz, 2H), 4.34 (br s, 1H), 7.31 (d, *J* = 8.1 Hz, 2H), 7.75 (d, *J* = 8.1 Hz, 2H); ^13^C NMR (75 MHz, CDCl_3_) δ 17.94, 21.29, 25.34, 27.59, 28.75, 42.80, 68.35, 84.05, 127.06, 129.90, 136.85, 143.34; Anal. calcd for C_14_H_19_NO_2_S: C, 63.36; H, 7.22; N, 5.28; found: C, 63.29; H, 7.16; N, 5.21.

## Supporting Information

File 1 Experimental procedures and NMR spectra for **4a**–**o** and **5a**, **b**, **g**–**m**, **o**, **6a**, **n**, **7**, **8**, **9**, **10**.
