# Supplementary material for: Intramolecular hydroamination of alkynic sulfonamides catalyzed by a gold–triethynylphosphine complex: Construction of azepine frameworks by 7-exo-dig cyclization
Source: Beilstein J Org Chem. 2011 Jul 8;7:951–9. doi: 10.3762/bjoc.7.106 (PMC3167492; doi:10.3762/bjoc.7.106)
Supplement: File 1 — Experimental procedures and NMR spectra for 4a–o and 5a, b, g–m, o, 6a, n, 7, 8, 9, 10. [file Beilstein_J_Org_Chem-07-951-s001.pdf]

Supporting Information

for

Intramolecular hydroamination of alkynic sulfonamides catalyzed by a gold–triethynylphosphine complex: construction of azepine frameworks by 7-*exo-dig* cyclization

Hideto Ito, Tomoya Harada, Hirohisa Ohmiya and Masaya Sawamura*

Address: Department of Chemistry, Faculty of Science, Hokkaido University, Sapporo 060-0810, Japan

Email: Masaya Sawamura* - sawamura@sci.hokudai.ac.jp

* Corresponding author

Experimental procedures and NMR spectra for 4a–o and 5a, b, g–m, o, 6a, n, 7, 8, 9, 10.

Table of contents

1. General……………………………………………………………………………………...S2
2. Time–conversion profiles for the gold-catalyzed cyclization of 4a with L1, X-Phos and IPr ligands……………………………………………………………………………… ….S2
3. Preparation of substrates……………………………………………………………… ……S3
4. Cyclization products………………………………………………………………… …..S7
5. References……………………………………………………………………………….S10

1. General

NMR spectra were recorded on a Varian Gemini 2000 spectrometer, operating at 300 MHz for ^{1}H NMR, 75.4 MHz for ^{13}C NMR and 121.4 MHz for ^{31}P NMR. Chemical shift values for ^{1}H, ^{13}C and ^{31}P NMR are referenced to Me_4Si, the residual solvent resonances and external aqueous 85% H_3PO_4 respectively. Mass spectrometry (JEOL JMS-FABmate for EI-MS, JEOL JMS-700TZ for ESI-MS) and elemental analysis were performed at the Instrumental Analysis Division, Equipment Management Center, Creative Research Institution, Hokkaido University. Triethynylphosphine ligand **L1** was prepared according to the reported procedure [1]. $AgNTf_2$ was prepared from Ag_2O and $HN(SO_2CF_3)_2$ [2]. $AgSbF_6$, $AgBF_4$ and AgOTf were purchased from Aldrich. Phosphine ligands, PPh_3 and $P(OPh)_3$ were commercially available. Gold complexes [AuCl(ligand)] were synthesized by the reported method.[1] MS4A was purchased from Junsei Chemical Co. Anhydrous solvents used in the synthesis of materials were purchased from Kanto Chemical Co. and used without further purification. Anhydrous CH_2Cl_2 and DCE were purchased from Kanto Chemical Co. and Aldrich, respectively, and these were degassed and dried with MS4A before use. Gel permeation chromatography (GPC) was performed by LC-908 (Japan Analytical Industry Ltd., two in-line JAIGEL-2H, $CHCl_3$, 3.5 mL/min, UV and RI detectors). TLC analyses were performed on commercial glass plates bearing 0.25-mm layer of Merck Silica gel $60F_{254}$. Silica gel (Kanto Chemical Co., Silica gel 60 N, spherical, neutral) was used for column chromatography. PTLC purification was performed on commercial glass plates bearing 1-mm layer of Merck Silica gel $60F_{254}$. All reactions were carried out under argon atmosphere unless otherwise noted.

2. Time–Conversion Profiles for the Gold-Catalyzed Cyclization of 4a with L1, X-Phos and IPr Ligands

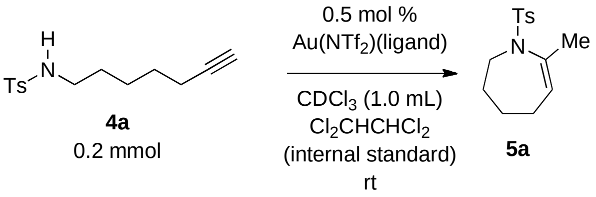

[$Au(NTf_2)$(**L1**)] (2.6 mg, 1.0 μmol, 0.5 mol %) was placed in a NMR tube equipped with a screw cap. Separately, the alkynyl sulfonamide **4a** (55 mg, 0.20 mmol) was weighed into a micro tube. The tubes were placed in a glove box. The gold complex and **4a** were dissolved in degassed dry $CDCl_3$ (0.25 mL), in their respective tubes. Then $Cl_2CHCHCl_2$ as an internal standard was added to a solution of the gold complex. A solution of **4a** was transferred to a solution of the catalyst with a syringe. The remaining solutions in the micro tube and the syringe were washed with CH_2Cl_2 (2 × 0.25 mL) and added to the reaction mixture. The tube was sealed with a cap equipped with a Teflon-coated silicon rubber septum. The tube was taken from the glove box, and was shaken at room temperature. The results with the corresponding ligands are shown in Table S1 and Figure S1.

Table S1: Time–Conversion/Yield Profiles for the Gold-Catalyzed Cyclization of 4a with L1, X-Phos and IPr Ligands.

			time							
entry	Au cat.		0.5 h	1 h	2 h	4 h	8h	20 h	38 h	58 h
1	$Au(NTf_2)$(DTBM-P)	conv.	6%	20%	45%	76%	99%	100%	-	-
		yield	6%	18%	40%	67%	88%	89%	-	-
2	$Au(NTf_2)$(IPr)	conv.	0%	0%	1%	7%	20%	43%	67%	88%
		yield	0%	0%	1%	7%	18%	38%	60%	84%
3	$Au(NTf_2)$(X-phos)	conv.	1%	6%	14%	25%	44%	73%	96%	100%
		yield	1%	5%	12%	22%	39%	65%	85%	88%

[a] Conversions and yields were determined by ^{1}H NMR

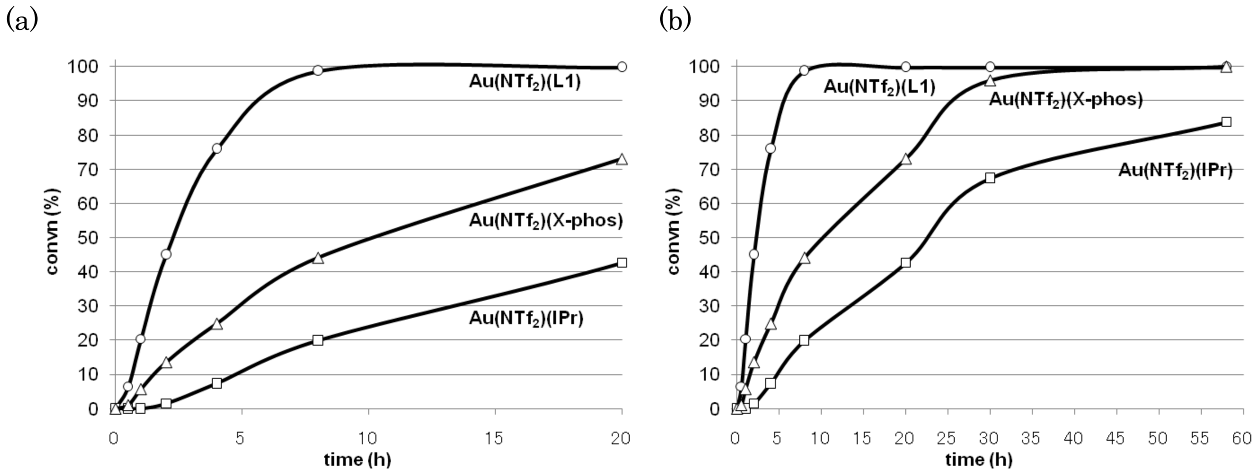

Figure S1: Time–Conversion Profiles for the Gold-Catalyzed Cyclization of 4a with L1, X-Phos and IPr Ligands. (a) the reactions between 0 to 20 h. (b) the reactions between 0 to 58 h.

3. Preparation of Substrates

Alkynyl sulfonamides **4a**, **4b** and alkynyl amine **4f** were prepared by the alkylation of $TsNH_2$, $NsNH_2$ or $PMBNH_2$ (2.0 eq) with the 7-iodo-1-trimethylsilyl-1-heptyne (1.0 eq) in the presence of $CsCO_3$ (1.2 eq) in DMF at 80 ˚C to 120 ˚C, followed by the deprotection of TMS group by K_2CO_3 (1.2 eq) in MeOH at rt.

Alkynyl carbamates **4c**, **4d** and **4e** were prepared by the protection of 6-heptyn-1-yl amine hydrochloride (1.0 eq) with *N*,*N*-dimethylaminopyridine (0.10 eq), Et_3N (3.0 eq) and CbzCl or Ac_2O or $(Boc)_2O$ (2.0 eq) in CH_2Cl_2 at rt.

α-Substituted alkynyl sulfonamides **4g**, **4h**, **4i** and **4o** were synthesized by the aziridine opening reactions of the corresponding *N*-tosyl aziridines (1.0 eq) with alkyl cuprates (1.5 eq) prepared from ^{t}BuLi (6.0 eq), 5-iodo-1-trimethylsilyl-1-pentyne (3.0 eq) and CuCN (1.5 eq) in THF at –78 ˚C, followed by the deprotection of TMS group by K_2CO_3 (1.2 eq) in MeOH at rt.

β-Disubstiuted alkynyl sulfonamides **4j**, **4k**, **4l** and benzyl sulfonamide **4m** were prepared by the reaction of the corresponding alkynyl amines (1.0 eq) with TsCl (1.5 eq), Et_3N (2.0 eq) and Me_3NHCl (0.1 eq) in CH_2Cl_2 at 0 ˚C. These amines were synthesized by the reductions of corresponding nitriles (1.0 eq) with $LiAlH_4$ (2.0 eq) in Et_2O at reflux.

N-Tosylbenzamide **4n** was prepared by the reaction of *N*-tosyl isocyanate (1.0 eq) and *o*-alkynyl benzoic acid (1.0 eq) in the presence of Et_3N (1.0 eq) in THF at reflux.

6a was synthesized by the reaction of *N*-tosyl ε-caprolactam (1.0 eq) with Cp_2TiMe_2 (5.0 eq) in toluene at 70 ˚C [3].

N-(6-Heptyn-1-yl)-4-toluenesulfonamide (4a)

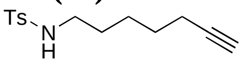

4a

White solid. Mp = 63.9–64.3 ˚C. ^{1}H NMR ($CDCl_3$) δ 1.32–1.53 (m, 6H), 1.94 (t, *J* = 2.7 Hz, 1H), 2.14 (td, *J* = 6.9, 2.7 Hz, 2H), 2.44 (s, 3H), 2.95 (q, *J* = 6.9 Hz, 2H), 4.34 (br s, 1H), 7.32 (d, *J* = 8.1 Hz, 2H), 7.75 (d, *J* = 8.1 Hz, 2H). ^{13}C NMR ($CDCl_3$) δ 17.95, 21.29, 25.34, 27.59, 28.75, 42.80, 68.35, 84.05, 127.06, 129.68, 136.85, 143.34. Anal. Calcd for $C_{14}H_{19}NO_2S$: C, 63.36; H, 7.22; N, 5.28%. Found: C, 63.27; H, 7.27; N, 5.27%.

N-(6-Heptyn-1-yl)-2-nitrobenzenesulfonamide (4b)

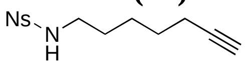

4b

Pale yellow solid. Mp = 70.9–72.7 ˚C. ^{1}H NMR ($CDCl_3$) δ 1.36–1.62 (m, 6H), 1.94 (t, *J* = 2.7 Hz, 1H), 2.16 (td, *J* = 6.6, 2.7 Hz, 2H), 3.12 (q, *J* = 6.6 Hz, 2H), 5.27 (br s, 1H), 7.72–7.80 (m, 2H), 7.84–7.92 (m, 1H), 8.11–8.19 (m, 1H). ^{13}C NMR ($CDCl_3$) δ 18.01, 25.33, 27.59, 28.94, 43.57, 68.51, 83.98, 125.45, 131.10, 132.88, 133.65, 133.70, 147.82. Anal. Calcd for $C_{13}H_{16}N_2O_4S$: C, 52.69; H, 5.44; N, 9.45%. Found: C, 52.62; H, 5.30; N, 9.42%.

Benzyl 6-heptyn-1-ylcarbamate (4c)

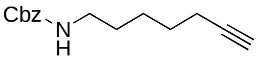

4c

Colorless oil. ^{1}H NMR ($CDCl_3$) δ 1.36–1.60 (m, 6H), 1.94 (t, *J* = 2.7 Hz, 1H), 2.19 (td, *J* = 6.6, 2.7 Hz, 2H), 3.20 (q, *J* = 6.3 Hz, 2H), 4.75 (br s, 1H), 5.10 (s, 2H), 7.28–7.39 (m, 5H). ^{13}C NMR ($CDCl_3$) δ 18.09, 25.58, 27.82, 29.28, 40.76, 66.48, 68.38, 84.21, 126.96, 128.10, 128.52, 136.64, 156.47. HRMS (ESI$^+$) Calcd for $C_{15}H_{19}NO_2Na$ $[M+Na]^+$: *m/z* 268. 13066. Found: *m/z* 268.13080.

N-(6-Heptyn-1-yl)-acetamide (4d)

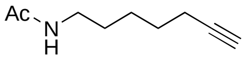

4d

Colorless oil. ^{1}H NMR ($CDCl_3$) δ 1.30–1.62 (m, 6H), 1.95 (t, *J* = 2.7 Hz, 1H), 1.98 (s, 3H), 2.20 (td, *J* = 6.6, 2.7 Hz, 2H), 3.26 (q, *J* = 6.6 Hz, 2H), 5.46 (br s, 1H). ^{13}C NMR ($CDCl_3$) δ 17.56, 22.28, 25.33, 27.40, 28.27, 38.76, 68.07, 83.63, 170.28. HRMS (ESI$^+$) Calcd for $C_9H_{15}NONa$ $[M+Na]^+$: *m/z* 176.10459. Found: *m/z* 176.10457.

***tert*-Butyl 6-heptyn-1-ylcarbamate (4e)**

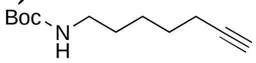

4e

Colorless oil. ^{1}H NMR ($CDCl_3$) δ 1.44 (s, 9H), 1.38–1.66 (m, 6H), 1.95 (t, *J* = 2.7 Hz, 1H), 2.20 (td, *J* = 6.9, 2.7 Hz, 2H), 3.12 (q, *J* = 6.9 Hz, 2H), 4.52 (br s, 1H). ^{13}C NMR ($CDCl_3$) δ 18.10, 25.66, 27.87, 28.23, 29.40, 40.27, 68.29, 78.89, 84.22, 156.03. HRMS (ESI$^+$) Calcd for $C_{12}H_{21}NO_2Na$ $[M+Na]^+$: *m/z* 234.14645. Found: *m/z* 234.14614.

N-(6-Heptyn-1-yl)-4-methoxybenzylamine (4f)

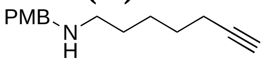

4f

Colorless oil. ^{1}H NMR ($CDCl_3$) δ 1.31 (br s, 1H), 1.36–1.62 (m, 6H), 1.94 (t, *J* = 2.4 Hz, 1H), 2.15–2.50 (m, 2H), 2.62 (t, *J* = 6.9 Hz, 2H), 3.72 (s, 2H), 3.80 (s, 3H), 6.87 (d, *J* = 8.4 Hz, 2H), 7.24 (d, *J* = 8.4 Hz, 2H). ^{13}C NMR ($CDCl_3$) δ 18.12, 26.26, 28.16, 29.38, 49.01, 53.30, 55.65, 68.14, 84.40, 113.68, 129.23, 132.65, 158.59. HRMS (ESI$^+$) Calcd for $C_{15}H_{22}NO$ $[M+H]^+$: *m/z* 232.16959. Found: *m/z* 232.16932.

(*S*)-*N*-(7-Octyn-2-yl)-4-toluenesulfonamide (4g)

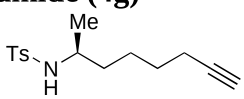

4g

White solid. Mp = 79.8–81.0 ˚C. ^{1}H NMR ($CDCl_3$) δ 1.03 (d, *J* = 6.6 Hz, 3H), 1.20–1.46 (m, 6H),

1.93 (t, J = 2.7 Hz, 1H), 2.09 (td, J = 6.6, 2.7 Hz, 2H), 2.43 (s, 3H), 3.25–3.36 (m, 1H), 4.18 (d, J = 8.1 Hz, 1H), 7.30 (d, J = 8.4 Hz, 2H), 7.76 (d, J = 8.4 Hz, 2H). ^{13}C NMR ($CDCl_3$) δ 18.05, 21.39, 21.57, 24.42, 27.82, 36.74, 49.72, 68.35, 84.17, 127.09, 129.71, 138.24, 143.32. Anal. Calcd for $C_{15}H_{21}NO_2S$: C, 64.48; H, 7.58; N, 5.01%. Found: C, 63.27; H, 7.79; N, 4.99%.

(*R*)-*N*-(2-Methyl-8-nonyn-3-yl)-4-toluenesulfonamide (4h)

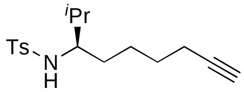

4h

White solid. Mp = 61.5–63.8 ˚C. ^{1}H NMR ($CDCl_3$) δ 0.79 (d, J = 6.9 Hz, 6H), 1.10–1.45 (m, 6H), 1.66–1.82 (m, 1H), 1.92 (t, J = 2.7 Hz, 1H), 2.04 (td, J = 6.9, 2.7 Hz, 2H), 2.43 (s, 3H), 3.03–3.13 (m, 1H), 4.25 (br s, 1H), 7.29 (d, J = 8.1 Hz, 2H), 7.76 (d, J = 8.1 Hz, 2H). ^{13}C NMR ($CDCl_3$) δ 17.46, 18.03, 18.18, 21.40, 24.60, 27.99, 31.08, 59.03, 68.34, 84.17, 127.11, 129.64, 138.57, 143.24. HRMS (ESI$^+$) Calcd for $C_{17}H_{25}NO_2SNa$ [M+Na]$^+$: *m/z* 330.14982. Found: *m/z* 330.14923.

(*R*)-*N*-(1-Phenyl-7-octyn-2-yl)-4-toluenesulfonamide (4i)

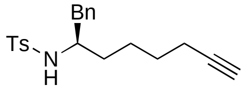

4i

White solid. Mp = 49.6–51.7 ˚C. ^{1}H NMR ($CDCl_3$) δ 1.20–1.52 (m, 6H), 1.92 (t, J = 2.4 Hz, 1H), 2.06 (td, J = 6.3, 2.4 Hz, 2H), 2.42 (s, 3H), 2.68 (d, J = 6.3 Hz, 2H), 3.42 (sextet, J = 6.9 Hz, 1H), 4.22 (d, J = 8.1 Hz, 1H), 6.98–7.03 (m, 2H), 7.19–7.30 (m, 5H), 7.66 (d, J = 8.4 Hz, 2H). ^{13}C NMR ($CDCl_3$) δ 18.05, 21.39, 24.29, 27.81, 33.62, 41.16, 54.67, 68.37, 84.19, 126.67, 127.07, 128.59, 129.55, 129.70, 137.06, 137.81, 143.30. HRMS (ESI$^+$) Calcd for $C_{21}H_{25}NO_2SNa$ [M+Na]$^+$: *m/z* 378.14982. Found: *m/z* 378.14953.

***N*-(2,2-Dimethyl-6-heptyn-1-yl)-4-toluenesulfonamide (4j)**

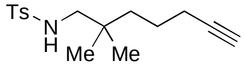

4j

White solid. Mp = 74.3–75.0 ˚C. ^{1}H NMR ($CDCl_3$) δ 0.85 (s, 6H), 1.23–1.33 (m, 2H), 1.33–1.45 (m, 2H), 1.97 (t, J = 2.7 Hz, 1H), 2.12 (td, J = 6.9, 2.7 Hz, 2H), 2.44 (s, 3H), 2.69 (d, J = 6.9 Hz, 2H), 4.37 (br s, 1H), 7.32 (d, J = 8.4 Hz, 2H), 7.74 (d, J = 8.4 Hz, 2H). ^{13}C NMR ($CDCl_3$) δ 18.68, 21.27, 22.64, 24.63, 33.43, 38.19, 52.56, 68.32, 84.34, 127.00, 129.65, 136.89, 143.23. Anal. Calcd for $C_{16}H_{23}NO_2S$: C, 65.49; H, 7.90; N, 4.77%. Found: C, 65.37; H, 7.93; N, 4.75%.

***N*-(2,2-Diphenyl-6-heptyn-1-yl)-4-toluenesulfonamide (4k)**

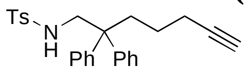

4k

White solid. Mp = 150.0–152.2 ˚C. ^{1}H NMR ($CDCl_3$) δ 1.06–1.18 (m, 2H), 1.91 (t, J = 2.7 Hz, 1H), 2.03 (td, J = 7.2, 2.7 Hz, 2H), 2.15–2.24 (m, 2H), 2.43 (s, 3H), 3.55 (d, J = 6.6 Hz, 2H), 3.85 (t, J = 6.6 Hz, 1H), 7.04–7.09 (m, 4H), 7.17–7.32 (m, 8H), 7.62 (d, J = 8.4 Hz, 2H). ^{13}C NMR ($CDCl_3$) δ 18.46, 21.32, 22.77, 35.46, 49.23, 49.28, 68.47, 83.94, 126.63, 127.06, 127.63, 128.39, 129.71, 136.29, 143.48, 144.77. HRMS (ESI$^+$) Calcd for $C_{26}H_{27}NO_2SNa$ [M+Na]$^+$: *m/z* 440.16547. Found: *m/z* 440.16518.

N-[{1-(4-Pentyn-1-yl)cyclohexyl}methyl]-4-toluenesulfonamide (4l)

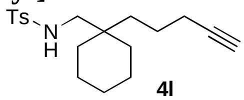

4l

White solid. Mp = 85.8–87.5 ˚C. ^{1}H NMR ($CDCl_3$) δ 1.16–1.52 (m, 14H), 2.00 (t, J = 2.7 Hz, 1H), 2.11 (td, J = 6.6, 2.7 Hz, 2H), 2.44 (s, 3H), 2.75 (d, J = 6.9 Hz, 2H), 4.36 (t, J = 6.9 Hz, 1H), 7.32 (d, J = 8.4 Hz, 2H), 7.75 (d, J = 8.4 Hz, 2H). ^{13}C NMR ($CDCl_3$) δ 18.81, 21.07, 21.42, 21.68, 25.92, 33.36, 33.96, 35.57, 48.88, 68.54, 84.62, 127.17, 129.78, 136.93, 143.43. Anal. Calcd for $C_{19}H_{27}NO_2S$: C, 68.43; H, 8.16; N, 4.20%. Found: C, 68.26; H, 8.43; N, 4.22%.

N-{2-(3-Butyn-1-yl)benzyl}-4-toluenesulfonamide (4m)

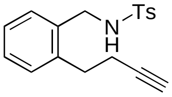

4m

White solid. Mp = 89.8–90.2 ˚C. ^{1}H NMR ($CDCl_3$) δ 1.98 (t, J = 2.7 Hz, 1H), 2.44 (td, J = 7.2, 2.7 Hz, 2H), 2.45 (s, 3H), 2.75 (t, J = 7.2 Hz, 2H), 4.13 (d, J = 5.7 Hz, 2H), 4.63 (br s, 1H), 7.13–7.31 (m, 4H), 7.34 (d, J = 8.1 Hz, 2H), 7.79 (d, J = 8.1 Hz, 2H). ^{13}C NMR ($CDCl_3$) δ 19.86, 21.41, 30.30, 44.75, 69.50, 83.58, 126.94, 127.29, 128.49, 129.71, 129.77, 129.77, 129.82, 133.81, 136.49, 138.91, 143.66. Anal. Calcd for $C_{18}H_{19}NO_2S$: C, 68.98; H, 6.11; N, 4.47%. Found: C, 68.99; H, 6.07; N, 4.60%.

2-(3-Butyn-1-yl)-N-tosylbenzamide (4n)

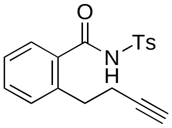

4n

White solid. Mp = 101.0–104.0 ˚C. ^{1}H NMR ($CDCl_3$) δ 1.96 (t, J = 2.4 Hz, 1H), 2.41 (td, J = 6.9, 2.4 Hz, 2H), 2.46 (s, 3H), 2.86 (t, J = 6.9 Hz, 2H), 7.20–7.33 (m, 2H), 8.03 (d, J = 8.4 Hz, 2H). The peak of N-H (1H) was not found. ^{13}C NMR ($CDCl_3$) δ 20.10, 21.56, 31.51, 69.70, 83.54, 126.82, 127.56, 128.51, 129.68, 131.21, 131.76, 132.51, 135.49, 139.83, 145.28, 166.45. Anal. Calcd for $C_{18}H_{17}NO_3S$: C, 66.03; H, 5.23; N, 4.28%. Found: C, 66.08; H, 5.32; N, 4.34%.

N-{*trans*-2-(4-Pentyn-1-yl)cyclohexyl}-4-toluenesulfonamide (4o)

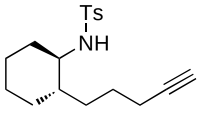

4o

White solid. Mp = 117.0–117.9 ˚C. ^{1}H NMR ($CDCl_3$) δ 0.85–1.21 (m, 7H), 1.22–1.38 (m, 1H), 1.39–1.53 (m, 1H), 1.54–1.70 (m, 2H), 1.74–1.87 (m, 2H), 1.94 (t, J = 2.7 Hz, 1H), 1.98–2.08 (m, 2H), 2.43 (s, 3H), 2.68–2.84 (m, 1H), 4.19 (d, J = 8.7 Hz, 1H), 7.30 (d, J = 8.1 Hz, 2H), 7.76 (d, J = 8.1 Hz, 2H). ^{13}C NMR ($CDCl_3$) δ 18.35, 21.32, 24.78, 24.89, 25.16, 30.49, 31.18, 34.28, 42.26, 56.96, 68.18, 84.45, 126.97, 129.59, 138.57, 143.11. HRMS (ESI$^+$) Calcd for $C_{18}H_{25}NO_2SNa$ $[M+Na]^+$: m/z 342.14982. Found: m/z 342.14962.

N-(5-Hexyn-1-yl)-4-toluenesulfonamide (7)

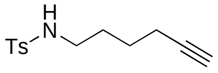

7

White solid. Mp = 61.8–63.5 ˚C. ^{1}H NMR ($CDCl_3$) δ 1.46–1.68 (m, 4H), 1.93 (t, J = 2.4 Hz, 1H),

2.16 (td, *J* = 6.6, 2.4 Hz, 2H), 2.45 (s, 3H), 2.97 (q, *J* = 6.6 Hz, 2H), 4.45 (br s, 1H), 7.32 (d, *J* = 8.1 Hz, 2H), 7.75 (d, *J* = 8.4 Hz, 2H). ^{13}C NMR ($CDCl_3$) δ 17.61, 21.31, 25.01, 28.23, 42.44, 68.67, 83.70, 127.06, 129.71, 136.80, 143.39. Anal. Calcd for $C_{13}H_{17}NO_2S$: C, 62.12; H, 6.82; N, 5.57%. Found: C, 62.16; H, 6.88; N, 5.54%.

_N_-(7-Octyn-1-yl) -4-toluenesulfonamide (9)

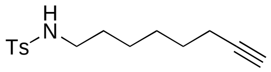

9

White solid. Mp = 52.8–54.2 ˚C. ^{1}H NMR ($CDCl_3$) δ 1.20–1.40 (m, 4H), 1.42–1.52 (m, 4H), 1.93 (t, *J* = 2.7 Hz, 1H), 2.15 (td, *J* = 6.9, 2.7 Hz, 2H), 2.94 (q, *J* = 6.9 Hz, 2H), 4.37 (br s, 1H), 7.32 (d, *J* = 8.1 Hz, 2H), 7.75 (d, *J* = 8.1 Hz, 2H). ^{13}C NMR ($CDCl_3$) δ 18.11, 21.41, 25.85, 27.97, 28.04, 29.33, 43.07, 68.29, 84.39, 127.18, 129.78, 137.05, 143.50. Anal. Calcd for $C_{15}H_{21}NO_2S$: C, 64.48; H, 7.58; 5.01%. Found: C, 64.49; H, 7.61; 5.02%.

4. Cyclization Products

7-Methyl-1-tosyl-2,3,4,5-tetrahydro-1*H*-azepine (5a)

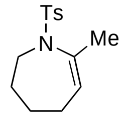

5a

White solid. Mp = 65.8–66.1 ˚C. ^{1}H NMR ($CDCl_3$) δ 1.30–1.40 (m, 2H), 1.73 (quint, *J* = 5.7 Hz, 2H), 1.84 (q, *J* = 6.3 Hz, 2H), 1.96 (s, 3H), 2.42 (s, 3H), 3.41 (t, *J* = 5.4 Hz, 2H), 5.51 (t, *J* = 6.9 Hz, 1H), 7.28 (d, *J* = 8.7 Hz, 2H), 7.77 (d, *J* = 8.7 Hz, 2H). ^{13}C NMR ($CDCl_3$) δ 21.14, 21.78, 23.53, 25.86, 29.49, 49.38, 126.36, 126.84, 129.27, 138.89, 139.33, 142.87. Anal. Calcd for $C_{14}H_{19}NO_2S$: C, 63.36; H, 7.22; N, 5.28%. Found: C, 63.29; H, 7.16; N, 5.21%.

7-Methyl-1-nosyl-2,3,4,5-tetrahydro-1*H*-azepine (5b)

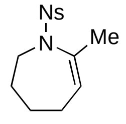

5b

Pale yellow solid. Mp = 93.7–95.7 ˚C. ^{1}H NMR ($CDCl_3$) δ 1.41–1.52 (m, 2H), 1.77–1.90 (m, 2H), 1.83 (s, 3H), 2.01–2.11 (m, 2H), 3.54 (t, *J* = 5.4 Hz, 2H), 5.63 (t, *J* = 6.6 Hz, 1H), 7.61–7.77 (m, 3H), 8.03–8.08 (m, 1H). ^{13}C NMR ($CDCl_3$) δ 21.70, 23.59, 26.27, 30.51, 50.48, 124.12, 127.91, 129.99, 131.68, 133.47, 135.13, 138.72, 148.21. Anal. Calcd for $C_{13}H_{16}N_2O_4S$: C, 52.69; H, 5.44; N, 9.45%. Found: C, 52.44; H, 5.29; N, 9.30%.

(*S*)-2,7-Dimethyl-1-tosyl-2,3,4,5-tetrahydro-1*H*-azepine (5g)

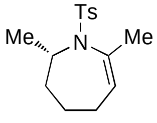

5g

White solid. Mp = 101.2–102.5 ˚C. ^{1}H NMR ($CDCl_3$) δ 1.11 (d, *J* = 7.2 Hz, 3H), 1.28–1.42 (m, 2H), 1.46–1.58 (m, 1H), 1.70–1.91 (m, 3H), 1.96 (s, 3H), 2.42 (s, 3H), 4.18–4.31 (m, 1H), 5.58–5.68 (m, 1H), 7.28 (d, *J* = 7.8 Hz, 2H), 7.78 (d, *J* = 8.1 Hz, 2H). ^{13}C NMR ($CDCl_3$) δ 17.01, 18.24, 21.38, 24.04, 26.35, 34.72, 51.91, 127.19, 127.97, 129.52, 134.85, 139.17, 142.94. Anal. Calcd for $C_{15}H_{21}NO_2S$: C, 64.48; H, 7.58; N, 5.01%. Found: C, 64.48; H, 7.82; N, 4.81%.

(*R*)-2-Isopropyl-7-methyl-1-tosyl-2,3,4,5-tetrahydro-1*H*-azepine (5h)

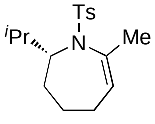

5h

White solid. Mp = 108.1–109.0 ˚C. ^{1}H NMR ($CDCl_3$) δ 0.84 (d, *J* = 6.6 Hz, 3H), 0.88 (d, *J* = 6.3 Hz, 3H), 1.13–1.34 (m, 2H), 1.64–1.74 (m, 2H), 1.75–1.90 (m, 2H), 2.04 (s, 3H), 2.42 (s, 3H), 3.60–3.69 (m, 1H), 5.54–5.61 (m, 1H), 7.26 (d, *J* = 8.1 Hz, 2H), 7.77 (d, *J* = 8.1 Hz, 2H). ^{13}C NMR ($CDCl_3$) δ 18.93, 19.47, 21.40, 21.54, 24.03, 26.94, 28.68, 30.65, 64.00, 127.51, 128.27, 129.36, 135.12, 138.82, 142.90. HRMS (EI$^+$) Calcd for $C_{17}H_{25}NO_2S$ [M]$^+$: *m/z* 307.16060. Found: *m/z* 307.16042.

(*R*)-2-Benzyl-7-methyl-1-tosyl-2,3,4,5-tetrahydro-1*H*-azepine (5i)

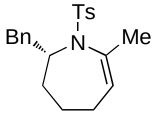

5i

Colorless viscous oil. ^{1}H NMR ($CDCl_3$) δ 1.31–1.44 (m, 2H), 1.44–157 (m, 1H), 1.62–1.84 (m, 1H), 1.84–1.96 (m, 1H), 1.99 (s, 3H), 2.42 (s, 3H), 2.77–2.85 (m, 1H), 4.28–4.38 (m, 1H), 5.63–5.72 (m, 1H), 7.05–7.10 (m, 2H), 7.17–7.30 (m, 5H), 7.73 (d, *J* = 8.3 Hz, 2H). ^{13}C NMR ($CDCl_3$) δ 18.58, 21.39, 24.03, 26.40, 31.18, 37.77, 57.89, 126.40, 127.29, 128.00, 128.51, 129.08, 129.52, 135.01, 138.60, 138.76, 143.04. HRMS (ESI$^+$) Calcd for $C_{21}H_{25}NO_2SNa$ [M+Na]$^+$: *m/z* 378.14982. Found: *m/z* 378.14954.

3,3,7-Trimethyl-1-tosyl-2,3,4,5-tetrahydro-1*H*-azepine (5j)

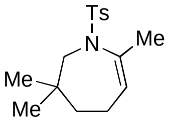

5j

White solid. Mp = 62.9–64.4 ˚C. ^{1}H NMR ($CDCl_3$) δ 0.99 (s, 6H), 1.16–1.23 (m, 2H), 1.75 (s, 3H), 2.05 (q, *J* = 6.0 Hz, 2H), 2.42 (s, 3H), 3.17 (s, 2H), 5.38 (t, *J* = 6.3 Hz, 1H), 7.27 (d, *J* = 8.1 Hz, 2H), 7.75 (d, *J* = 8.1 Hz, 2H). ^{13}C NMR ($CDCl_3$) δ 20.56, 21.37, 21.72, 26.94, 34.64, 37.65, 59.37, 125.73, 127.08, 129.50, 139.36, 139.83, 142.95. Anal. Calcd for $C_{16}H_{23}NO_2S$: C, 65.49; H, 7.90; N, 4.77%. Found: C, 65.41; H, 7.96; N, 4.78%.

3,3-Diphenyl-7-methyl-1-tosyl-2,3,4,5-tetrahydro-1*H*-azepine (5k) and 2-Methylene-6,6-diphenyl-1-tosylazepane (6k) [5k/6k = 92/8]

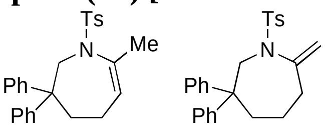

5k **6k**

White solid. Mp = 152.1–152.4 ˚C. ^{1}H NMR of **5k** ($CDCl_3$) δ 1.94 (br s, 5H), 2.31–2.41 (m, 2H), 2.39 (s, 3H), 4.40 (s, 2H), 5.15 (t, *J* = 4.8 Hz, 1H), 7.10–7.30 (m, 12H), 7.35 (d, *J* = 8.4 Hz, 2H) {**6k**: δ 4.16 (s, 2H), 4.69 (s, 1H), 4.84 (s, 1H), 7.65 (d, *J* = 8.1 Hz, 2H)}. ^{13}C NMR of **5k** ($CDCl_3$) δ 21.37, 22.31, 23.65, 33.14, 49.64, 57.71, 125.14, 126.03, 126.91, 127.83, 128.08, 129.33, 137.29, 138.51, 142.73, 147.30. HRMS (EI$^+$) Calcd for $C_{26}H_{27}NO_2S$ [M]$^+$: *m/z* 471.17625. Found: *m/z* 417.17611.

9-Methyl-8-tosyl-8-aza-9-spiro[5.6]dodecene (5l)

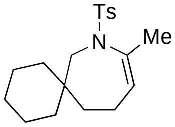

5l

White solid. Mp = 73.6–74.8 ˚C. ^{1}H NMR ($CDCl_3$) δ 1.22–1.50 (m, 12H), 1.77 (s, 3H), 1.97–2.08 (m, 2H), 2.42 (s, 3H), 3.27 (s, 2H), 5.35 (t, *J* = 6.6 Hz, 1H), 7.27 (d, *J* = 8.1 Hz, 2H), 7.54 (d, *J* = 8.1 Hz, 2H). ^{13}C NMR ($CDCl_3$) δ 20.76, 21.04, 21.28, 21.37, 26.29, 34.06, 35.12, 36.40, 59.11, 125.44, 127.00, 129.49, 139.13, 139.80, 142.91. Anal. Calcd for $C_{19}H_{27}NO_2S$: C, 68.43; H, 8.16; N, 4.20%. Found: C, 68.45; H, 8.37; N, 4.18%.

3-Methyl-2-tosyl-2,5-dihydro-1*H*-benzo[*c*]azepine (5m)

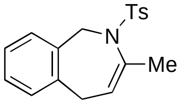

5m

White solid. Mp = 109.0–112.8 ˚C. ^{1}H NMR ($CDCl_3$) δ 2.07 (s, 3H), 2.30 (s, 3H), 3.31 (br d, *J* = 3.6 Hz, 2H), 4.82 (s, 2H), 5.18–5.24 (m, 1H), 6.77 (d, *J* = 6.9 Hz, 1H), 6.98 (d, *J* = 8.1 Hz, 2H), 7.06–7.21 (m, 4H), 7.25 (d, *J* = 8.4 Hz, 1H). ^{13}C NMR ($CDCl_3$) δ 21.27, 23.86, 33.96, 54.11, 118.65, 126.33, 127.14, 127.68, 128.93, 129.27, 129.33, 135.76, 136.93, 137.33, 137.79, 142.60. Anal. Calcd for $C_{18}H_{19}NO_2S$: C, 68.98; H, 6.11; N, 4.47%. Found: C, 69.04; H, 6.26; N, 4.47%.

3-Methylene-2-tosyl-2,3,4,5-tetrahydro-1*H*-1-benzo[*c*]azepinone (6n)

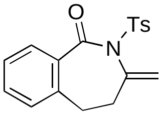

6n

White solid. Mp = 126.0–128.1 ˚C. ^{1}H NMR ($CDCl_3$) δ 2.45 (s, 3H), 2.94 (br s, 4H), 5.09 (s, 1H), 5.14 (s, 1H), 7.13 (d, *J* = 7.5 Hz, 1H), 7.24–7.32 (m, 1H), 7.35–7.43 (m, 3H), 7.64 (dd, *J* = 7.8, 1.2 Hz, 1H), 8.08 (*J* = 8.4 Hz, 2H). ^{13}C NMR ($CDCl_3$) δ 21.59, 29.95, 37.84, 120.57, 127.32, 128.92, 129.25, 129.57, 129.92, 132.61, 133.42, 136.05, 138.88, 141.97, 145.15, 168.36. HRMS (ESI^{+}) Calcd for $C_{18}H_{17}NO_3SNa$ $[M+Na]^{+}$: *m/z* 350.08214. Found: *m/z* 350.08177.

***trans*-2-Methyl-1-tosyl-4,5,5a,6,7,8,9,9a-octahydro-1*H*-benzo[*b*]azepine (5o) and *trans*-2-methylene-1-tosyldecahydro-1*H*-benzo[*b*]azepine (6o) [5o/6o = 98/2]**

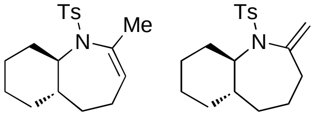

5o **6o**

White solid. Mp = 106.6–109.9 ˚C. ^{1}H NMR of **5o** ($CDCl_3$) δ 0.85–1.44 (m, 9H), 1.56–2.05 (m, 4H), 2.06 (s, 3H), 2.42 (s, 3H), 3.25–3.36 (m, 2H), 5.40–5.46 (m, 1H), 7.25 (d, *J* = 8.4 Hz, 2H), 7.76 (d, *J* = 8.4 Hz, 2H) {**6o** δ 4.83 (s, 1H), 5.17 (s, 1H)}. ^{13}C NMR of **5o** ($CDCl_3$) δ 21.41, 23.88, 25.24, 25.99, 26.15, 27.64, 33.84, 35.30, 40.51, 65.15, 126.99, 127.18, 129.43, 133.18, 138.88, 142.89. Anal. Calcd for $C_{18}H_{25}NO_2S$: C, 67.67; H, 7.89; N, 4.38%. Found: C, 67.76; H, 8.14; N, 4.17%.

2-Methyleneazepane (6a)

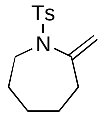

6a

Pale yellow oil. This compound slowly decomposed to the polar unidentified materials at room temperature. ^{1}H NMR ($CDCl_3$) δ 1.44–1.56 (m, 4H), 1.69–1.80 (m, 2H), 2.05–2.13 (m, 2H), 2.42 (s, 3H), 3.51–3.57 (m, 2H), 4.82 (s, 1H), 4.98 (s, 1H), 7.28 (d, *J* = 8.4 Hz, 2H), 7.71 (d, *J* = 8.4 Hz, 2H). ^{13}C NMR ($CDCl_3$) δ 21.31, 27.71, 27.94, 28.38, 34.63, 48.86, 110.03, 127.41, 129.39, 136.72, 143.20, 146.13. HRMS (ESI^+) Calcd for $C_{14}H_{19}NO_2SNa$ $[M+Na]^+$: *m/z* 288.10287. Found: *m/z* 288.10312.

6-Methyl-1-tosyl-1,2,3,4-tetrahydropyridine (7)

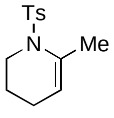

8

White solid. Mp = 77.9–78.9 ˚C. ^{1}H NMR ($CDCl_3$) δ 1.33–1.43 (m, 2H), 1.86–1.92 (m, 2H), 2.11 (br q, *J* = 1.8 Hz, 3H), 2.43 (s, 3H), 3.59–3.65 (m, 2H), 5.00 (br s, 1H), 7.29 (d, *J* = 8.1 Hz, 2H), 7.68 (d, *J* = 8.1 Hz, 2H). ^{13}C NMR ($CDCl_3$) δ 20.22, 21.30, 22.33, 22.94, 46.71, 113.64, 126.91, 129.62, 134.07, 137.53, 143.26. HRMS (ESI^+) Calcd for $C_{14}H_{19}NO_2SNa$ $[M+Na]^+$: *m/z* 288.10287. Found: *m/z* 288.10312. Anal. Calcd for $C_{13}H_{17}NO_2S$: C, 62.12; H, 6.82; N, 5.57%. Found: C, 62.18; H, 6.87; N, 5.54%.

(*E*)- or (*Z*)-8-Methyl-1-tosyl-1,2,3,4,5,6-hexahydroazocine (10)

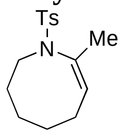

10

White solid. *E*/Z configuration was not determined. Mp = 80.3–80.8 ˚C. ^{1}H NMR ($CDCl_3$) δ 1.43–1.65 (m, 6H), 1.68 (s, 3H), 2.10–2.19 (m, 2H), 2.42 (s, 3H), 3.30 (t, *J* = 4.8 Hz, 2H), 5.71 (td, *J* = 8.1, 1.2 Hz, 1H), 7.29 (d, *J* = 8.4 Hz, 2H), 7.76 (d, *J* = 8.4 Hz, 2H). ^{13}C NMR ($CDCl_3$) δ 18.45, 21.38, 25.91, 26.93, 26.97, 28.80, 49.18, 127.37, 129.59, 133.04, 133.81, 138.41, 143.06. Anal. Calcd for $C_{15}H_{21}NO_2S$: C, 64.48; H, 7.58; N, 5.01%. Found: C, 64.51; H, 7.61; N, 5.01%.

5. Reference

[1] Ochida, A.; Ito, H.; Sawamura, M. *J. Am. Chem. Soc.* **2006**, *128*, 16486–16487.

[2] Williams, D. B; Stoll, M. E.; Scott, B. L.; Costa, D. A.; Oldham, W. J. Jr. *Chem. Commun.* **2005**, 1438–1440.

[3] Martínez, I.; Howell, A. R. *Tetrahedron Lett.* **2000**, *41*, 5607–5611.
